# I CAN Intervention to Increase Grit and Self-Efficacy: A Pilot Study

**DOI:** 10.3390/brainsci14010033

**Published:** 2023-12-29

**Authors:** Hermundur Sigmundsson, Håvard Hauge

**Affiliations:** 1 Department of Psychology, Norwegian University of Science and Technology, 7491 Trondheim, Norway; havard.hauge@ntnu.no; 2 Research Center for Education and Mindset, University of Iceland, 102 Reykjavik, Iceland

**Keywords:** I CAN, assessment, Grit, Growth Mindset, Self-Efficacy, Well-Being, learning, achievement

## Abstract

In recent years, there has been a growing interest in increasing motivational factors within the domain of psychology. Among these factors, Grit, Mindset, Self-Efficacy, and Well-Being (Flourishing) have been suggested to play an important role in individuals’ performance and Well-Being. Thus, cultivating these factors in the general population is important. Previous interventions have displayed substantial effects in certain areas. However, these interventions have primarily been Mindset oriented. This paper presents a novel intervention approach by also emphasizing the importance of brain development; the importance of stimuli for building a network in the brain; the importance of repetition for strengthening the network; and the importance of perseverance and deliberate practice for achievement. The purpose of the current study was to examine the effects of a 35–40 min online intervention to increase the beliefs of ‘I CAN’ for 38 university students in Norway. The mean age of the 38 participants was 22.55 (SD = 1.59) and they completed a pre-test assessment of the Grit-S Scale, Theories of Intelligence Scale (Mindset), General Self-Efficacy Scale, and Flourishing Scale (Well-Being). This was followed up by the novel intervention and finally a post-test of the scales eight weeks later. The results showed an increase in Grit, Self-Efficacy, and Well-Being. However, only Grit displayed a significant increase. We aimed at creating an intervention where the participants would “turn on the switch”, meaning that they develop stronger beliefs. These promising results warrant a further development of the intervention, and studies with a larger group.

## 1. Introduction

It is well established that our brain has the capacity to change, even in late adulthood [1,2,3]. However, plasticity is still related to age. Grey and white matter density declines with age in most cortices [4], and the areas related to executive functions and memory are especially vulnerable [5]. The young, developing brain has higher sensitivity to reward and more glutamate compared to adults [6], which is beneficial for long-term potentiation. Frank et al. [7] also found that children’s inhibitory processes (GABA fluctuations) stabilize faster after visual tasks than in adults, making them more adept to learn larger quantities of information within a given time period. 

The Norwegian School system is based on regular knowledge acquirement. That is, the learning objectives are predominantly met when knowledge about certain topics is acquired by students. Although knowledge of this kind is important, there are some arguments as to why time at school should also be allocated to activities beyond traditional teaching. Technological advances suggest that the future job market will be more unstable [8], leading to more stressors for future students to handle. Studies also suggest that mental disorders are on the rise for adolescents, especially after COVID-19 [9]. In addition, a recent study found that 22% of Norwegian adolescents are categorized as anxious [10]. Thus, there seems to be a present and future need to allocate resources on adolescents’ Well-Being in the classroom. An evidence-based review [11], discussing important skills for the 21-century, has suggested that motivation, Grit, and Self-Efficacy (among others) are critical factors to thrive today and in the coming times. We concur with this idea and posit that the interventions implemented within schools, aiming to enhance these factors, could serve as a suitable strategy to promote the Well-Being of adolescents.

Psychological interventions have received a substantial amount of attention, especially Mindset-oriented ones. A well-recognized meta-analysis revealed that these interventions only seem to have a positive effect on academic performance on students with a low socioeconomic background [12]. A large, national US Mindset intervention with over 12,000 students also found that Mindset interventions mainly benefited lower-achieving students, but also increased enrolment rates to advances math course among students [13] (p. 364). 

A more recent Mindset intervention, which combined a growth Mindset approach with a “stress as adaptive” protocol, also yielded promising results [14]. Burnette et al. [15] conducted a recent meta-analysis and found that Mindset interventions have moderate health-, performance-, and social functioning effects, but point to challenges to draw clear intervention guidelines due to heterogenous effects. To this date, there is no consensus regarding Mindset intervention approaches. Our novel intervention places emphasis on developmental theories, including important learning principles and research on Grit, Growth Mindset, Self-Efficacy, and Well-Being [16]. 

## 2. Theoretical Background

Neural group selection theory is a neurobiological theory explaining how we develop from birth and adapt to internal and external stimuli throughout life [17]. Thus, the theory may be used as a lens to understand how individuals learn and develop. Just a few weeks after birth, our brain begins generating neurons which allows the brain to form neuronal groups through a probabilistic process. This lays the foundation of different brain parts which Edelman calls the primary repertoire [18]. This repertoire makes it possible to develop and strengthen (or weaken) neuronal groups depending on stimuli [19]. Both external and internal stimuli play a crucial role by carving out frequently activated pathways, making them pronounced and efficient. The pathways not exposed to any stimuli eventually decay [19,20]. Thus, it is crucial for children and adolescents to regularly be exposed to the knowledge or competency that are beneficial to maintain, whether they are of a social or technical nature. In this respect, Kleim and Jones [21] (p. 227) argue to ‘use it or lose it’ and ‘use it and improve it’. 

The development of knowledge: Ericsson et al. [22] suggested that to acquire solid skills or competence in an area, a large amount of practice hours is necessary, which is easy to advocate for. However, his real contribution derives from how these hours should be spent practicing. He coined the term ‘deliberate practice’ to explain that high competence within a certain field requires specific, effortful, and goal-targeted practice. In addition, the importance of immediate feedback and the guidance of a coach has been emphasized [23]. Guidance from someone who knows how it should be done and feedback to learn if the mental representations work or need to be adjusted are, according to Ericsson, crucial. At the same time, passion [16], Grit [24], and Mindset [13] are of great importance for achievement. 

Drawing on Edelman and Ericsson’s work, specificity has been emphasized in the intervention. There is solid evidence suggesting that competency in one area does not generalize, even within the same domain [25,26,27,28,29]. Thus, to enhance constructs such as Self-Efficacy [30], the intervention attempts to create a feeling of “I can”, and preferably equip students with strategies allowing for the repetition of this feeling in upcoming situations, which will strengthen neuronal pathways related to Self-Efficacy [16]. 

Comparing to skill and knowledge development, which need lot of training and repetition to be excellent [17,18,19,22,27], beliefs can be developed like turning on a switch [13]. The neurological explanation could be increased focus, i.e., enhanced gamma synchrony [31], the greater activation of the amygdala (emotional processing) [32], or dopamine activity [33]. Vander Weele et al. [34] (p. 1) argue that: “dopamine may underlie a diversity of functions by modulating the signal -to-noise ratio in subpopulations of mPFC neurons”.

## 3. Constructs

In our research group ‘Learning and skill development’, we are focusing on motivational factors, i.e., passion for achievement [16], Grit/perseverance [24], Mindset/Growth Mindset [35], and Self-Efficacy [30]. In addition, we are focusing on flow [36,37] and Well-Being (Flourishing) [38]. When developing our intervention, we built on the research we conducted over the last 28 years, with main emphasis on the theory of Edelman (neural Darwinism), Ericsson (deliberate practice), Duckworth (Grit), and Dweck (Mindset). We know from research that these factors are important for Self-Efficacy and achievement. We also know that both Grit and Mindset have been found to be important to Well-Being. Therefore, we chose to measure the following in this pilot study: Grit, Mindset, Self-Efficacy, and Well-Being (Flourishing). 

**Grit** has been defined in the literature as perseverance and passion towards long-term goals [24]. It is positively associated with academic achievement [39], student motivation [40], as well as the amount of time spent on deliberate practice [41]. Students higher in Grit have also been observed to be more positive towards learning and open to constructive criticism [42].

**Mindset** refers to the implicit beliefs we have about ourselves and others [13,43]. Mindset has been shown to influence academic achievement and Mindset interventions to be beneficial for students from lower socioeconomic backgrounds [12].

**Self-Efficacy** has been defined by Bandura [44] (p. 3) as ‘‘beliefs in one’s capabilities to organize and execute the courses of action required to produce given attainments’’. Students with high levels of Self-Efficacy tend to try harder, endure longer, choose more demanding challenges, experience these efforts more positively, and feel less anxiety, compared to those with low Self-Efficacy [45]. 

**Well-Being** (Flourishing) is synonymous with a high level of mental Well-Being, epitomizes mental health, and has been suggested to play an important part for adolescents’ daily quality of life [46,47]. 

## 4. The Current Study

In the present study, we aim to investigate a new intervention with the main focus on participants’ knowledge of brain development; the importance of stimuli for building a network in the brain [19]; the importance of repetition for strengthening the network [21]; and the importance of perseverance [24] and deliberate practice for achievement [23]. In this article, we report the development of an intervention which has the main aim to increase the knowledge of the factor mentioned and the feeling of ‘I CAN’ among the individuals who take the intervention. It will say to increase the participants’ Grit, Growth Mindset, Self-Efficacy, and Well-Being. 

## 5. Method

### 5.1. Sample

In total, 38 subjects participated in the study. The average age in the group was 22.55 (SD = 1.59). The average age of the female group was 23.36 (SD = 1.74, *n* = 14), and the male group had an average age of 22.08 (SD = 1.32; *n* =24). All the participants answered the survey. The participants were randomly selected among university students in Trondheim, Norway. Participants indicated their gender, age, and educational level. 

### 5.2. Measurements

**Grit**: A Norwegian version of the Short Grit Scale (Grit-S) was used to measure Grit [48]. The eight-item scale is thought to be a more cost- and time-effective version of the original Grit scale and has been shown to have reliable psychometric features [49]. The Likert scale ranges from 1 (“Not me at all”) to 5 (“Very typical me”). The scale consists of two subscales: “perseverance of effort” and “consistency of interest”. The latter subscale has received criticism for lacking incremental validity [39,50]. “Perseverance of effort” tries to capture the hard-working mentality of individuals reaching long-term goals (e.g., “I finish whatever I start”). The remaining four items are intended to gauge long-term (constant) interest in general (e.g., “new ideas and projects sometimes distract me from previous ones”). All the items are reversed on this subscale. For this study, the Cronbach’s α of 0.92 was found for the pre-test and 0.83 for the post-test.

**Mindset**: A Norwegian version of Dweck’s [51] Theories of Intelligence Scale (TIS) was used to assess students’ entity and incremental conceptions of intelligence [16,52]. The self-form for adults of this measure was used to ensure that the students focused on their ideas about their own intelligence (and not their ideas about people in general). This scale consists of several subscales with items rated on a six-point Likert-type scale, from 1 (Strongly Agree) to 6 (Strongly Disagree). The items included differ between those associated with an incremental theory (i.e., Growth Mindset) and those associated with an entity theory (i.e., Fixed Mindset). The reliability data for the scale come from Dweck et al. [43] and are based on the eight-item scale. The scale shows good internal consistency (α = 0.85) and test-retest reliability at 2 weeks (r = 0.80). The scale also shows a good construct validity with the scores predicting a meaningful relationship with several variables [43]. The Norwegian version of TIS has also shown to be reliable, with Cronbach’s α of 0.86 for entity items and 0.88 for the incremental items [52]. For this study, the Cronbach’s α of 0.94 was found for the pre-test and 0.94 for the post-test.

**Self-Efficacy**: To measure Self-Efficacy, the General Self-Efficacy Scale was used [53]. The 10-item scale has been extensively used in research and assesses individuals’ own belief in dealing with novel or difficult challenges, including setbacks and obstacles in life. The scale uses a four-point Likert scale ranging from “not at all true” to “exactly true” and has demonstrated good internal consistency and construct validity, also across cultures. For this study, the Cronbach’s α of 0.82 was found for the pre-test and 0.89 for the post-test. 

**Well-Being**: The Flourishing Scale [38] was deployed to evaluate students’ Well-Being. Diener et al. [38] describes the brief, eight-item scale as a self-evaluation of important life areas such as optimism, self-esteem, and relationships, and thus, a single psychological construct which encapsulates Well-Being. The scale uses a seven-point Likert scale ranging from “strongly disagree” to completely agree”, and has been found to have good psychometric properties, also across different samples and among adolescents [47,54,55]. For this study, the Cronbach’s α of 0.97 was found for the pre-test and 0.96 for the post-test. 

### 5.3. Procedure

The study was performed in accordance with the Declaration of Helsinki. Because the study did not collect sensitive personal data, passive consent from the participants was confirmed to be sufficient from the Norwegian Centre for research data (NSD). The information registered about the participants was anonymous (only age and gender). To be able to couple the pre- and post-date, the participants used a code. The data collection was carried out by trained research assistants and conducted on http://nettskjema.no.

The interventions did not ask for any sensitive data; thus, no application was sent to the Norwegian Data protection Authority. It was made clear to all participants that their participation was optional and that they could withdraw at any moment. Lastly, the participants were informed that both interventions included a post-test 8 weeks after the intervention, only including the scales given at the start of the intervention. 

Study design: it was a study with three parts in the study: a pre-test, intervention, and post-test eight week later. 

The first part of the intervention (pre-test) included demographic variables (age, gender, occupation), the Grit-S, Implicit theories of intelligence, The Passion scale, The Flourishing Scale, and The General Self Efficacy Scale. The post-test included the Grit-S, Implicit theories of intelligence, The Passion scale, The Flourishing Scale, and The General Self Efficacy Scale.

### 5.4. Intervention I CAN

The intervention placed an emphasis on:

(1) How the brain’s structure and malleability make an optimal foundation for learning and development;
(2) The importance of effort and long-term commitment in increasing their skill/knowledge and becoming an expert;
(3) Mindset and having a “not yet” mentality, recognizing that additional training is essential to further refine their skills and master future challenges;
(4) The importance of passion—defined as a strong interest in an area/theme/skill. We highlighted that passion is an important motivational force providing necessary focus to achieve long-term goals. Therefore, we encouraged students to spend time on things they enjoy to develop their passion(s);
(5) The intervention concludes by prompting participants to reflect on their learning experience by creating a scenario where they offer advice (written form) to fellow students struggling with a difficult subject. Specific examples of malleability were given, such as how sustained effort over time enlarged taxi drivers’ hippocampal volume [56]. An example of how deliberate practice can lead to extraordinary results was also illustrated. We used Ericsson and Pool’s [23] example of how an ordinary university student went from being able to recall 7 digits to 82 digits, using deliberate practice.

### 5.5. Data Analysis

The data were analyzed in SPSS (version 27). We used paired sampled *t*-test to analyze the differences between the pre- and post-test for the four factors. Statistical significance was set at *p* < 0.05.

## 6. Results

Table 1 presents the score for the pre- and post-test for Grit, Mindset, Self-Efficacy, and Well-Being. The results indicate a higher score for the post-test for three of the four factors: grit (24.35 vs. 26.57), Self-Efficacy (31.63 vs. 32.01), and Well-Being (47.64 vs. 48.62). However, only the score for Grit was significant (*p* = 0.012). 

## 7. Discussion

The purpose of the current study was to examine the effects of a 35–40 min online intervention to increase the beliefs of ‘I CAN’ for 38 university students in Norway. We aimed at creating an intervention where the participants would “turn on the switch”, meaning that they develop stronger beliefs. By working with learning, the importance of focused training, and the importance of perseverance and courage, we can change attitudes and beliefs in individuals. We can increase the feeling of I CAN [57].

We found higher scores after the intervention related to Grit (from 24.37 to 26.57), Self-Efficacy (from 31.63 to 32.01), and Well-Being (from 47.64 to 48.62). However, the only significant finding was related to the score in Grit. This shift in beliefs may possibly be related to dopamine activity [58], the greater activation of the amygdala [32], or increased focus, i.e., enhanced gamma synchrony [31]. Earlier, Seligman and Maier [58] did argue that through experience, organisms form beliefs which may play an important role in their motivation [13] (p. 482). 

This is a very interesting finding despite the small number of participants (*n* = 38). The findings may be supported by the study of Yeager et al. [13] (*n* = 12.490), where they found a significant effect of the Growth Mindset intervention on academic performance in lower-achieving adolescents (14–15 years old). The main focus of their intervention was that intellectual abilities can be shaped and developed. As early as 1482, Giovanni Pico Della Mirandola claimed that humans are the only species with the ability to transform themselves, i.e., ‘their potential to make themselves into what they aspire to be’ [13] (p. 482). Dweck and Yeager [59] argue in their paper that Growth Mindset interventions may enhance motivation and Well-Being. It may be argued that the study of Riley [60] also supports our findings. She found the impact of the female role model on secondary school students’ exam performance in Uganda. The largest effect was found for female students. Riley [60] (p. 35) argued: ‘that schools should place more emphasis on having appropriate role models in schools, whether through showing a movie or through having former students come in to tell their stories. This should be particularly effective at closing the gender gaps and in helping the worst performing students’. 

Interesting in this respect is the perspective from Heider [61]. He argues that there are two factors which may have an influence on the outcome. One of the factors he mentions is ‘can’, which indicates whether the goals could be reached or not. This is closely related to the concept of the power and ability of the individual, which could be linked to both Grit [62] and Mindset [35]. The other factor is ‘trying’, or the motivation to try which may be linked to passion for achievement [16,48] and courage. So, building on Heider’s [61] view, we could argue that increased ‘I CAN’ beliefs may create more opportunities for a person to take on new challenges, increase courage, and provide more possibilities for finding passion and developing it [16]. In this line, it could be argued that individuals learn that their effort is very important for their success [63]. This is supported by Ericsson and Pool [23] who argue that ‘deliberate practice’ is key to achievement. 

Although Grit has received criticism [42,50,64], scholars still argue that the construct has substantial cultivation value [65,66,67]. In our post-test measurement, we found a significant increase in grit, which is especially exciting since there has been an ongoing debate as to whether grit could in fact be increased [68].

In regard to Grit and education, it has generally been believed that students who have a strong commitment to their studies and persevere in the face of academic and social problems are more likely to achieve academic success [66]. Indeed, a substantial part of research on Grit has been in relation to academic performance [39,50,69]. Previous studies have also demonstrated that Grit and mental health are connected. A recent review suggests that Grit is important for physical health and Well-Being outcomes, among other factors (See [70]). Thus, the implications of an increase in Grit may reach beyond academic performance among students. This is an important notion since mental distress is on the rise among youth in Norway [71]. 

One reason why we managed to increase Grit may be seen in light of one of the few studies (to the authors’ knowledge) which primarily aimed at increasing Grit [64]. Alan et al. [65] found an increase in Grit in the intervention group (compared to the control group) in a 2.5-year follow-up assessment in Turkish students. The present pilot intervention has a substantial content overlap as Alan et al. [65], such as (1) Mindset Theory, (2) the importance of effort in improving abilities and accomplishing objectives, and (3) the value of pursuing activates (including goals) of interest. Although we presented this content differently from Alan et al. [65], it may be that an increase in Grit was found because both studies included these three concepts in one intervention. However, it should be noted that the current pilot study and other interventions have only found modest post-measurement effects [65], and that the literature is too nascent to draw any clear conclusions as to how much Grit may be increased and how interventions should be developed.

## 8. Limitations and Future Research

A clear limitation of the study is the number of participants. Our research group is planning research with both adolescents (15–16 years old) and young adults (16–19 years), where the aim is to have many participants who will be randomly assigned to either an experimental group or a control group. We aim to include the same measures as in this pilot study: Grit, Mindset, Self-Efficacy, and Well-Being. 

## 9. Conclusions

The intervention increased the score in three of four variables, i.e., Grit, Self-Efficacy, and Well-Being. This pilot study is very promising. That 35–40 min online intervention is able to “turn on the switch”, meaning that the participants develop stronger beliefs or the feeling of I CAN. The results from this study will be followed by two larger studies carried out to get a better picture of this important issue.

## Figures and Tables

**Table 1:** Mean and Standard Deviations for pre-test and post-test for Grit, Self-efficacy, and Well-Being (*n* = 38).

	Pre-Test		Post-Test		
	Mean	SD	Mean	SD	*p* *
Grit	24.37	2.02	26.57	5.09	0.012
Mindset	35.47	6.86	35.38	7.01	0.447
Self-Efficacy	31.63	3.03	32.01	4.12	0.245
Well-Being	47.64	7.07	48.62	4.73	0.132

* Paired samples *t*-test.

## Data Availability

The datasets used and analyzed in the current study are available from the corresponding author upon reasonable request. That is the practice in our research group.
